# Supplementary material for: The Platelet Collagen Receptor GPVI Is Cleaved by Tspan15/ADAM10 and Tspan33/ADAM10 Molecular Scissors
Source: Int J Mol Sci. 2022 Feb 23;23(5):2440. doi: 10.3390/ijms23052440 (PMC8910667; doi:10.3390/ijms23052440)
Supplement: Supplementary file 1 [file ijms-23-02440-s001.zip › ijms-1596130-supplementary.pdf]

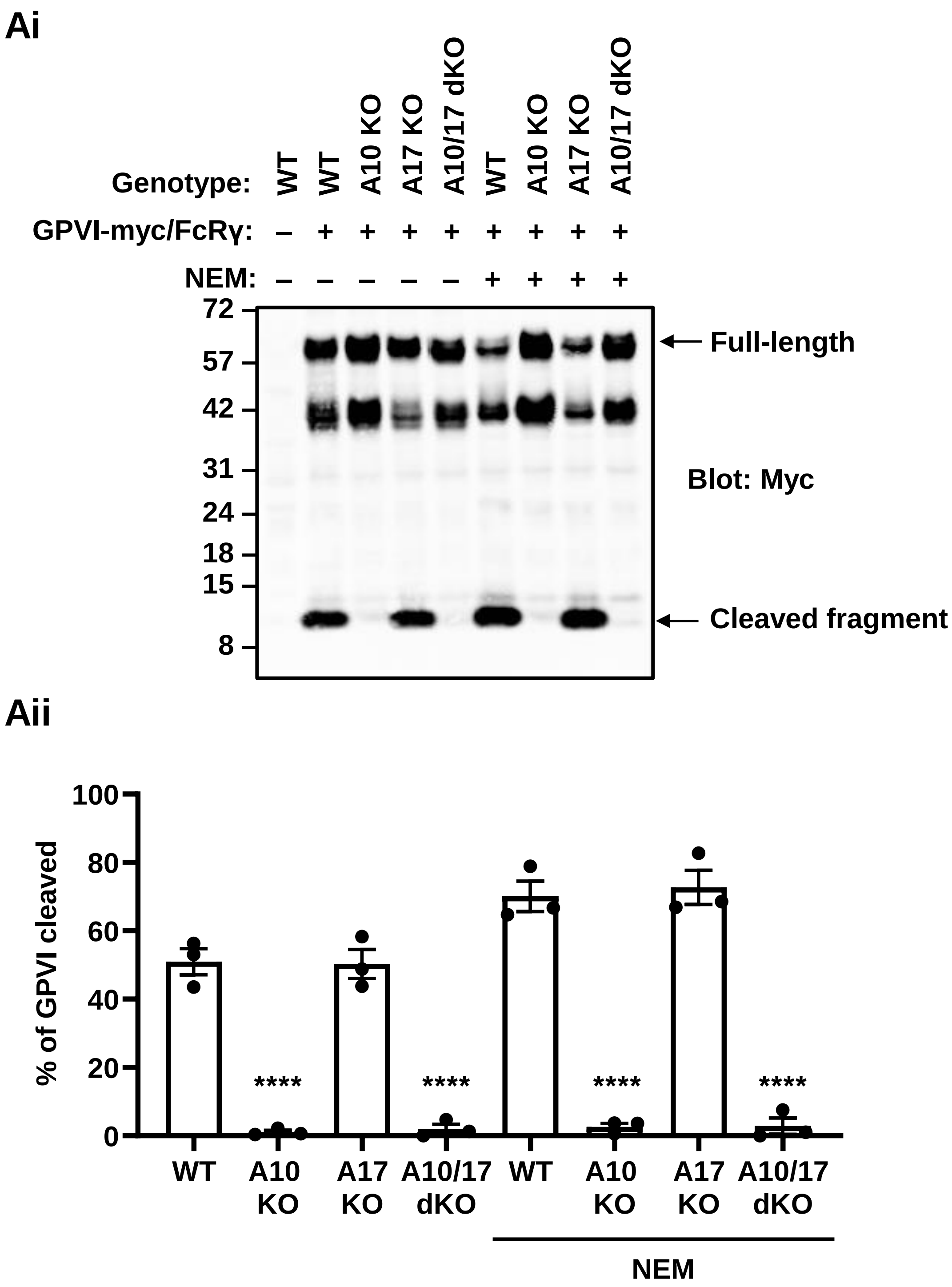

Supplementary Figure S1. Cleavage of GPVI in transfected HEK-293T cells is ADAM10-dependent. (Ai) Wild-type (WT), ADAM10-knockout (A10 KO), ADAM17-knockout (A17 KO), and ADAM10/17 double knockout (A10/17 dKO) HEK-293T cells were transfected with expression constructs for C-terminally myc-tagged human GPVI and FcRγ (+), or empty vector (–). Cells were treated with 2 mM NEM (+), or vehicle control (–) for 30 min prior to harvest. Cells were then lysed in 1% Triton X-100 and lysates subjected to Western blotting with an anti-myc antibody. (Aii) The percentage of cleaved GPVI from Ai was calculated. Error bars represent the standard error of the the mean from three independent experiments. Data were acrsine-transformed and statistically analyzed using a two-way ANOVA followed by a Dunnett's multiple comparison test, compared to their respective WT controls in each stimulation condition (****, $p < 0.0001$).

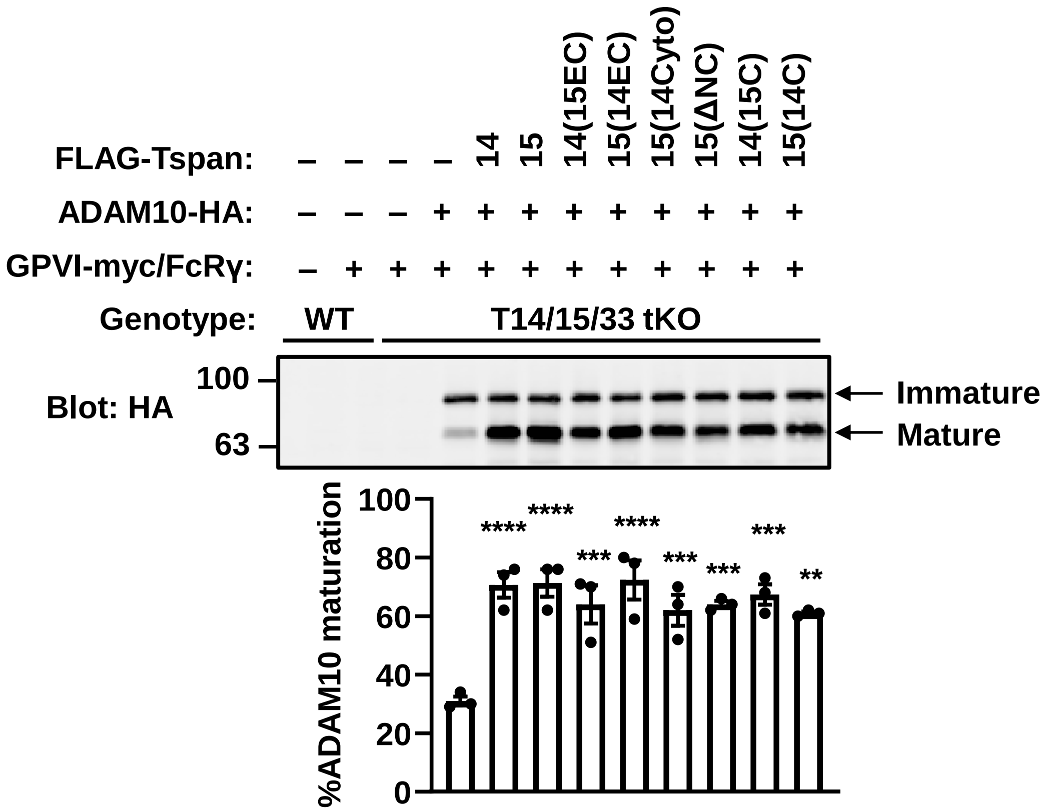

Supplementary Figure S2. Tspan14 and Tspan15 mutants support ADAM10 maturation. Cells and constructs were as described in Figure 4. The percentage of ADAM10 maturation was quantitated by expressing the smaller, mature ADAM10 as a percentage of total ADAM10 (mature and immature). Error bars represent the standard error of the the mean from three independent experiments. Data were acrsine-transformed and statistically analyzed using a one-way ANOVA followed by a Dunnett's multiple comparison test, compared to cells transfected with ADAM10 only (**, $p < 0.01$; ***, $p < 0.001$; ****, $p < 0.0001$).

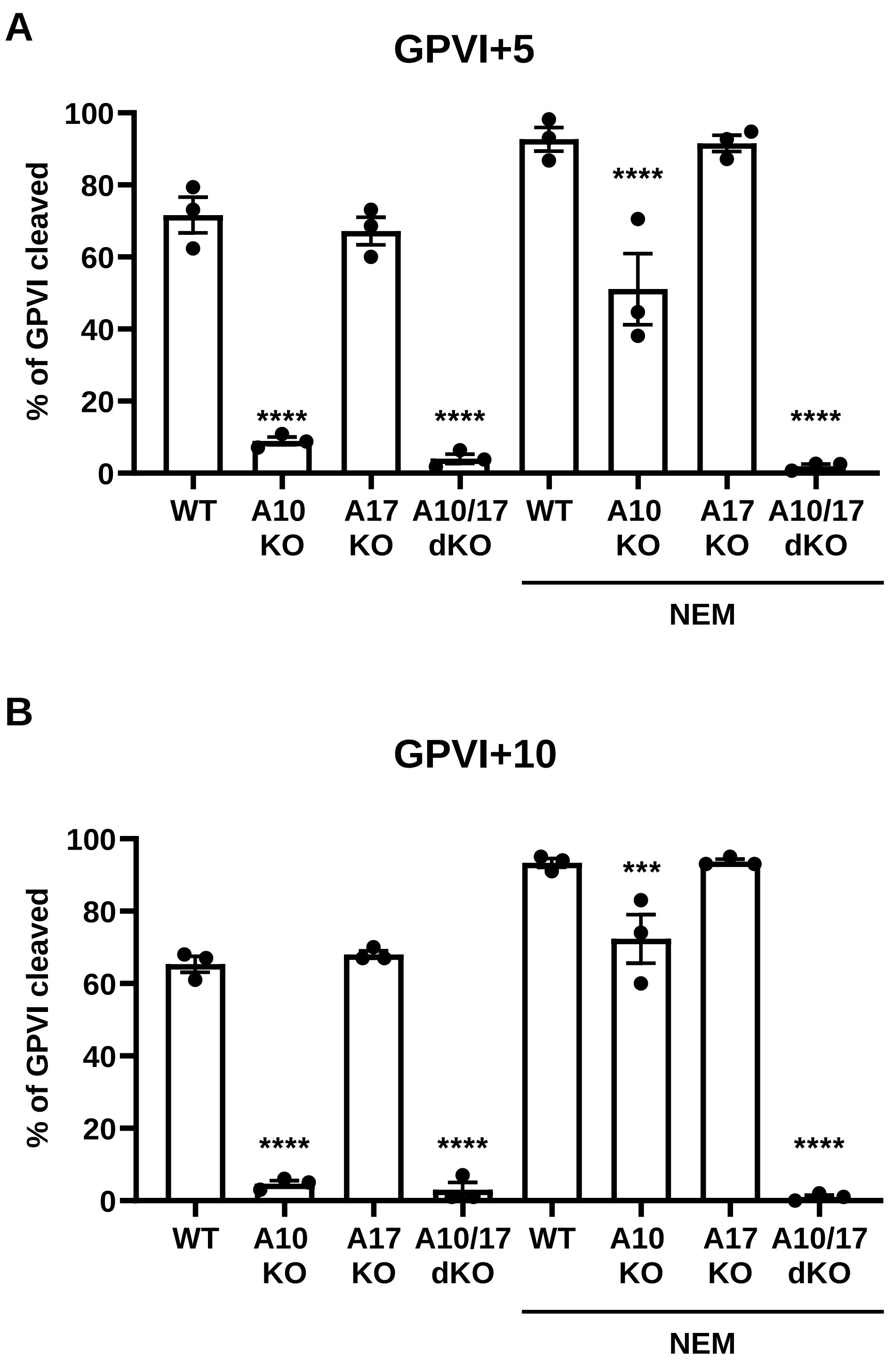

Supplementary Figure S3. ADAM17 can cleave stalk-extended GPVI mutant in the absence of ADAM10. Wild-type (WT), ADAM10-knockout (A10 KO), ADAM17-knockout (A17 KO), and ADAM10/17 double knockout (A10/17 dKO) HEK-293T cells were transfected with expression constructs for C-terminally myc-tagged stalk-extension mutants, (A) GPVI+5 or (B) GPVI+10 and FcRγ (+), or empty vector (–). Cells were treated with 2 mM NEM (+), or vehicle control (–) for 30 min prior to harvest. Cells were then lysed in 1% Triton X-100 and lysates subjected to Western blotting with an anti-myc antibody. The percentage of cleaved GPVI was calculated. Error bars represent the standard error of the the mean from three independent experiments. Data were acrsine-transformed and statistically analyzed using a two-way ANOVA followed by a Dunnett's multiple comparison test, compared to their respective WT controls in each stimulation condition (***, $p < 0.001$; ****, $p < 0.0001$).
